# Word Decoding of Protein Amino Acid Sequences with Availability Analysis: A Linguistic Approach

**DOI:** 10.1371/journal.pone.0050039

**Published:** 2012-11-21

**Authors:** Kenta Motomura, Tomohiro Fujita, Motosuke Tsutsumi, Satsuki Kikuzato, Morikazu Nakamura, Joji M. Otaki

**Affiliations:** 1 The BCPH Unit of Molecular Physiology, Department of Chemistry, Biology and Marine Science, University of the Ryukyus, Nishihara, Okinawa, Japan; 2 Department of Information Science, University of the Ryukyus, Nishihara, Okinawa, Japan; Max Planck Institute for the Physics of Complex Systems, Germany

## Abstract

The amino acid sequences of proteins determine their three-dimensional structures and functions. However, how sequence information is related to structures and functions is still enigmatic. In this study, we show that at least a part of the sequence information can be extracted by treating amino acid sequences of proteins as a collection of English words, based on a working hypothesis that amino acid sequences of proteins are composed of short constituent amino acid sequences (SCSs) or “words”. We first confirmed that the English language highly likely follows Zipf's law, a special case of power law. We found that the rank-frequency plot of SCSs in proteins exhibits a similar distribution when low-rank tails are excluded. In comparison with natural English and “compressed” English without spaces between words, amino acid sequences of proteins show larger linear ranges and smaller exponents with heavier low-rank tails, demonstrating that the SCS distribution in proteins is largely scale-free. A distribution pattern of SCSs in proteins is similar among species, but species-specific features are also present. Based on the availability scores of SCSs, we found that sequence motifs are enriched in high-availability sites (i.e., “key words”) and vice versa. In fact, the highest availability peak within a given protein sequence often directly corresponds to a sequence motif. The amino acid composition of high-availability sites within motifs is different from that of entire motifs and all protein sequences, suggesting the possible functional importance of specific SCSs and their compositional amino acids within motifs. We anticipate that our availability-based word decoding approach is complementary to sequence alignment approaches in predicting functionally important sites of unknown proteins from their amino acid sequences.

## Introduction

Anfinsen's dogma or thermodynamic hypothesis states that the amino acid sequences of proteins are necessary and sufficient to determine their three-dimensional structures and functions that realize kinetically probable and stable free energy minimum states [1]. Information extraction from amino acid sequences is thus a crucial step in understanding protein molecules. At present, structural and functional prediction from amino acid sequences largely depends on the intricate use of the accumulated experimental data in the Protein Data Bank (PDB) [2], together with the fundamental use of sequence alignments [3]. However, a general rule on how protein sequence information is related to three-dimensional structures and functions is largely unknown.

It has been known that many molecular biological programs for sequence analysis contain a relation with linguistics mostly implicitly [4]. But in some cases, explicit use of linguistic tools for biological sequences has been performed, especially for analysis of nucleotide sequences [4]–[6]. Linguistic applications to protein amino acid sequences are less frequent, but Hidden Markov models (HMMs), an application from speech processing, have been widely employed to analyze a wide variety of proteins from different viewpoints (e.g. [7], [8]). In other studies, secondary structure predictions based on linguistic rules, i.e., grammar, have been proposed [4], [9], [10]. These approaches are largely based on formal language theory. On the other hand, there is an approach based on so-called “literary linguistics” including stylistics and textual analysis [4].

In this study, we propose a novel approach based on literary linguistics that decodes the amino acid sequences of proteins using an analogy between amino acid sequences and a natural language, e.g., English. Our linguistic approach does not involve sophisticated algorithms. Instead, it is “intuitive” or “primitive”, meaning that we search for correspondence between short stretches of amino acid sequences and English words. Proteins are composed of the 20 “letters” of amino acids, whereas English sentences use the 26 letters of the alphabet. English sentences are also composed of an organized collection of words, and one could examine proteins based on a working hypothesis that amino acid sequences can be considered to be collections of short constituent sequences (SCSs), or “words”, such as triplets (three-amino-acid stretches), quartets (four-amino-acid stretches), and pentats (five-amino-acid stretches), that are meaningfully localized by a set of rules, i.e., “grammar”.

We previously demonstrated the importance of short stretches in proteins by showing that length of secondary structures peaked at 5 or 6 amino acids [11], [12], which could justify our protein analysis based on SCSs. Our SCSs are basically identical to *k*-tuples [13]–[16], but we consider SCSs more like real linguistic words for general decoding, not merely a collection of simple analysis units for alignment-free sequence comparisons. Another synonym is n-grams, and the n-gram analysis has recently been performed widely in extracting information from biological sequences [17]–[21]. To our knowledge, however, the direct and intuitive comparison between English and protein amino acid sequences has not been performed.

An empirical law called Zipf's law is known to be widely applicable to natural languages, including English. This law states that the occurrence or frequency of words in a system of natural language is inversely proportional to their ranks [22]. Zipf's law is a special case of a power function that is expressed as *y = a x^-b^*, where *x* is the rank, *y* is the frequency, *a* is a coefficient, and *b* is an exponent that is a positive rational number. In languages, the exponent is said to be nearly 1, in which case such a power function is called Zipf's law. This rank-frequency relationship in languages holds true over at least a few orders of magnitude, which can be shown by linear ranges of the log-log plot of *Y = A – bX* where *Y* = log *y*, *X*  = log *x*, and *A* = log *a*. This “scale-free” relationship appears to result from communication tradeoffs between the speaker and the hearer. The communication tradeoffs are known as the principle of least effort [23], [24], which drives the evolution of natural languages. Speakers try to minimize their verbal effort to convey a given idea, preferring brief and ambiguous words, whereas hearers want to minimize the process of understanding, preferring brief but unambiguous words.

This tradeoff relationship between speaker and hearer could be analogous to the relationship between the primary and tertiary structures of proteins. During evolution, the primary structure “wants to” minimize its mutational amino acid changes, preferring small sequential changes that cause only ambiguous functional changes, whereas the tertiary structure “needs” small changes that are precisely required for a particular function. This metaphorical expression is simply another way of saying that protein sequences result from molecular evolution driven by random and parsimonious changes of amino acid sequences and by subsequent natural selection for the stringent functionality of folded protein molecules. Therefore, there is a possibility that protein sequences evolved based on the principle of least effort and hence at least partially follow Zipf's law, or more generally, a power law. In this study, we examined whether the SCSs in proteins [11], [12], [25]–[27], which could be called “words”, exhibit a similar or dissimilar distribution to a power-law distribution.

We note that variations of SCSs are very large but exactly limited in number. For example, all possible three-letter combination of amino acids (i.e., triplet) numbers exactly 8,000 ( = 20^3^) and no more. Similarly, all possible four-letter and five-letter combinations (i.e., quartet and pentat) number exactly 160,000 ( = 20^4^) and 3,200,000 ( = 20^5^), respectively. It is possible, using modern computational power, to comprehensively document the occurrence of all possible combinations of *n* amino acids (i.e., SCSs) in a large protein database, if *n* is relatively small, and this documentation can be referred to as the “dictionary” of word occurrence. Our strategy is to examine word usage using a collection of non-redundant amino acid sequences (nr-aa) by referring to the dictionary of word occurrence. We defined the availability score (*A*) for each SCS in a given data set of letters as its relative occurrence (see Materials and Methods) [25]–[27]. Using the availability score as a tool for sequence examination, we further compared high-availability sites with known motifs using a collection of representative proteins from PDB-REPRDB [28]. Together, the present study shows that this linguistic approach is likely useful in decoding protein amino acid sequences, focusing on putative important sites, or “key words”, which are defined as high-availability sites.

## Materials and Methods

### Database and Program Resources

The entire nr-aa (non-redundant amino acid) database, which includes all known amino acid sequences, was downloaded on August 2, 2012 (ftp://ftp.ncbi.nlm.nih.gov/blast/db/FASTA/nr.gz). In the nr-aa database, a given sequence was recorded only once. When just a single amino acid is different between two sequences, they were recorded as independent entries. Thus, each nr-aa entry is supposed to be unique. This database amounted to 4,656,296,401 bytes (11,211,279,389 bytes after decompression), containing 113,382,218 lines and 19,630,785 sequences.

Species-specific databases were constructed by collecting sequences that have annotation of a given species name from the nr-aa database. The species-specific databases of human (*Homo sapiens*), fruit fly (*Drosophila melanogaster*), thale cress (*Arabidopsis thaliana*), and colon bacterium (*Escherichia coli*) contained 229,084 sequences, 43,208 sequences, 64,087 sequences, and 24,123 sequences, respectively.

For English sentences, the content of Wikipedia English version “20120802 No. 23” was downloaded on August 2, 2012 (http://dumps.wikimedia.org/enwiki/20120802/enwiki-20120802-pages-meta-current23.xml-p018225001p020925000.bz2). This database amounted to 1,098,053,588 bytes (5,845,167,428 bytes after decompression). This 1 GB file was a part of the entre 16.5 GB articles, but we considered it a representative of the entire article. The original data were written in XML, and thus they contained many control codes associated with XML and Wikimedia, which were deleted as follows. Article data were extracted using Parse::MediaWikiDump of Perl. HTML expressions and MediaWiki expressions were deleted using fgetss() and Text_Wiki_Mediawiki of PHP, respectively. We also deleted lines that begin with the following: “|”, “#”, “!”, “{”, and “}”. Lines that include “://” were also deleted to avoid URL expressions. Lines with less than 200 bytes were deleted. Lines with 4,096 bytes were deleted if they contained “{{”. A portion that was enclosed by {{ and }} were also deleted. After these operations, alphabets were extracted from the database, and lower-case letters were converted to upper-case letters. In the case of natural English, the number of words was counted by our own program written in C language. In producing the compressed English database, spaces between words were deleted.

As described in our previous studies [26], [27], we downloaded the structural files from the Research Collaboratory for Structural Bioinformatics Protein Data Bank [2] (RCSB-PDB or simply PDB in this paper; http://www.pdb.org/) on November 18–19, 2007. To avoid the redundant structural information, we focused on 1,590 entries (1,643 protein chains) of which the PDB-IDs were specified by the PDB-REPRDB [28] (http://mbs.cbrc.jp/pdbreprdb-cgi/). The PDB-REPRDB can specify a collection of representative PDB entries in which similar entries in terms of amino acid sequence and three-dimensional structure were eliminated. Thus, each PDB-REPRDB entry is supposed to be unique. Among the 1,644 protein chains specified, one sample was not found in the PDB, and two files had wrong specifications on secondary structures. These three files were eliminated from our analysis. Therefore, we subsequently analyzed 1,641 proteins [26], [27].

### Power Law Distributions

After recording the numbers and ranks of all possible words in a database, log[*rank*] (expressed as log*R*) and log[*number*] (expressed as log*N*) were calculated, and they were plotted on a log*R*-log*N* two-dimensional space. A power law *y = ax^-b^* in an *x-y* plane can be converted to *Y = A – bX* in the logarithmic *X-Y* space. Accordingly, a plot between log*R* and log*N* shows a straight line if a given collection of words follow a power law. The best-fit least-squares line was drawn and its associated *a* (intercept), *b* (slope or exponent), and *r* (correlation coefficient) were calculated using gnuplot 4.6 (www.gnuplot.info; Geeknet, Inc., Fairfax, VA, USA, 2010). The best-fit line was shown by a thick red line, and other thin lines were drawn in 0.05 exponent intervals.

After producing the *X-Y* plots, the linear ranges and their exponents were determined manually. To do this, the best-fit line of *Y* = *A* – *bX* that was the closest to a English or protein distribution was first determined. The linear range was defined as the range in which the distribution of interest did not cross the straight lines of ±0.15 exponents from the best-fit line. This definition was set to meet a requirement of achieving a linear width of more than two orders of magnitudes both in *X*- and *Y*-axes in natural English. This is a proposed requirement for a power-law distribution by Stumpf and Porter [29]. Based on this definition, linear range and linear width were determined for natural English, compressed English, and amino acid sequences.

For statistical evaluation for power-law distributions, a python program called powerlaw. 4.1 was used [30]–[32], which was downloaded in August 2012 at http://pypi.python.org/pypi/powerlaw#downloads. This program was run with Scientific Linux (containing numpy, atlas, and lapack), blas, and scipy. Discriminant *R* value was used to judge the feasibility of a given database to favor a power law. This discriminant *R* value is a likelihood ratio between power law and other distributions, in this case, an exponential. When a power law distribution is likely, discriminant *R* is positive, whereas when it is unlikely, discriminant *R* is negative.

Using these methods, we compared behaviors of natural English, “compressed” English, and protein amino acid sequences to describe how similar or dissimilar a protein distribution is to an English distribution, and we did not intend to obtain exact parameters of a probability density function for possible power-law fits.

### Calculation of Availability Scores

The basic definition and calculation of availability scores for doublets and triplets are described elsewhere [25]–[27]. Briefly, we defined the difference between the probabilistically estimated count *E* and the real count *R* for each doublet or triplet in a database as the relative count or availability for a given doublet or triplet. The availability score *A* can be expressed as follows:
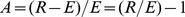
(1)


In this equation, *E* is calculated in the case of a triplet as follows:
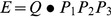
(2)where *Q* is the total number of existing triplets in a database and *P_1_*, *P_2_* and *P_3_* are the probabilities that a given amino acid appears at a position, which are derived from occurrence of each amino acid in that database. The probabilistically estimated count *E* does not consider influences from near-by amino acids and thus cannot be used singularly as an frequency indicator for real proteins. What we would like to know is a possible biological bias in amino acid sequences that can be extracted by availability score *A* that is given in Eq. (1). It is this bias that makes availability-based analysis valuable.

A more elaborate and accurate expression of *E*, which we used in calculating *A* in the present study, is given as follows.
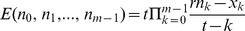
(3)


To explain this Eq. (3), we first define *a_k_* as the (*k+1*)th amino acid in an *m*-aa SCS (i.e., an amino acid *m*-mer). The other letters in this equation are defined as follows: *T*, total number of amino acids; *t*, total number of *m*-aa SCSs; *n_k_*, number of *a_k_*; and *x_k_*, number of *a_k_* in a set {*a_0_*, *a_1_*, *a_2_*, …, *a_k−1_*}. Additionally, *r* is defined as *t/T*.

Let us examine the process of calculating *E* of a pentat (5-amino-acid SCS), AAMAC. Here, *a_0_* = A (alanine), *a_1_* =  A, *a_3_* = M (methionine), *a_4_* = A, and *a_5_* = C (cysteine). Suppose that there are 180 As, 30 Cs, and 50 Ms in a database. Thus, *n_0_* = 180, *n_1_* = 180, *n_2_* = 50, *n_3_* = 180, and *n_4_* = 30.

Now, *x_k_* is the number of *a_k_* in a set {*a_0_*, *a_1_*, *a_2_*, …, *a_k−1_*}. For example, let us focus on the fourth amino acid of the pentat AAMAC, that is, *a_3_* = A. Here, we write a set {*a_0_*, *a_1_*, *a_2_*, …, *a_k−1_*} = {A, A, M}, and because *a_3_* = A is included twice in this set, *x_3_* = 2. This process is performed for all of the amino acids in AAMAC, and we obtain *x_0_* = 0, *x_1_* = 1, *x_2_* = 0, *x_3_* = 2, and *x_4_* = 0. Using these numbers, *E* can be calculated as follows.
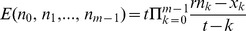


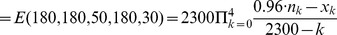


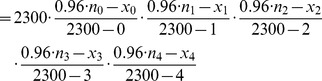


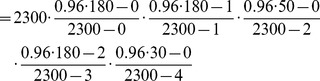


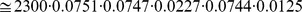


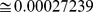



### Construction of Availability Database and Availability Plot Program

Sequences were extracted from the FASTA format database. Linefeed codes were deleted. Original description of a protein sequence often occupies two or more lines. We corrected it by assigning only one line per sequence. When non-standard amino acid codes (i.e., X, U, B, Z, J, O, and others) appeared, this sequence was considered to end at that point, and analysis was commenced from the next amino acid. The total number of amino acids and total number of *n*-aa SCSs in the collected sequences were calculated by our own analysis program written in C language. Similarly, the numbers of all possible *n*-aa SCSs (*n* = 1, 2, …, 6) in the collected sequences were calculated. Using these numbers, the availability scores were calculated for each *n*-aa SCS using Eq. (3), which resulted in the availability database. The availability plot program was constructed to assign availability score found in the availability database to each *n*-aa SCS. We used a MacPro computer with a CPU 2.53 GHz Intel core 2 Duo and 4 GB (1067 MHz) DDR memory or with a CPU 3 GHz 4 core Xenon x2 and 12 GB (667 MHz) memory for these calculations. Graphical output of the availability plot program was set to be drawn in JavaScript.

The availability plot program is open to the public as a web-interactive program, which is a part of the SCS package posted at http://bio.ads.ie.u-ryukyu.ac.jp, updated on September 9, 2012. The updated SCS package is based on the nr-aa and English Wikipedia databases downloaded on August 2, 2012. The original version of the availability plot program was based on the nr-aa database downloaded on November 25, 2009 and on the entire English Wikipedia on November 7, 2009, which is still available at http://bio.ads.ie.u-ryukyu.ac.jp/200902/. The availability analysis performed in the present study used the original version of the nr-aa and English Wikipedia databases. Users should note that this program accepts only a single sequence at a time for a manual query. Both original and relative availability scores are displayed in a table, but only relative availability values are shown graphically. Users are encouraged to use Microsoft Excel and other related software to make one's own availability plots using the spreadsheet-friendly outputs of this program. The SCS package is a web server that also contains other availability-based programs. These programs are already open to the public, but currently under evaluation and thus not yet formally published.

### Motif Analysis and Rank Order Analysis

Web-interactive PROSITE scan [33] (http://prosite.expasy.org/) was used for motif identification. We focused on the sequence motifs that contain 20 amino acids or less. Based on this length limit, we identified 521 motifs that contain 6,842 amino acids in 397 proteins out of 1,641. The average length of these motifs was 13.13 amino acids. Among these motifs, there were 195 motifs that contained high-availability sites (*A*≥3), which were analyzed in terms of amino acid composition. In contrast, there were 73 proteins out of 1641 that contain motifs longer than 21 amino acids.

To examine the correspondence between availability peaks and sequence motifs, we first defined the relative availability score (*rA*) in a given protein. The largest availability score was set to 100%, and the other scores were proportionally adjusted to yield relative scores in a given protein. We examined how well the 100% *rA* sites and the more-than-50% *rA* sites corresponded to sequence motifs. We obtained percentages of proteins in which there is a direct correspondence between motifs and those high *rA* sites.

The distribution of the availability scores of sequence motifs was compared with that of a random stretch of 13 amino acids the sequence of which was specified randomly in protein sequences that contain the identified motifs. When the random assignment process specified a 13-amino-acid stretch that overlapped with identified motifs, that 13-amino-acid stretch was discarded and the next random search was conducted until 521 random sequences were obtained. The rank order distances of the amino acid composition were calculated as a simple subtraction of the order number of the query sequences from those of the full sequences. The rank order distance (ROD) scores for each pool of amino acids, which indicates deviation in rank order from the full sequences, were calculated as described in the previous study [26]. The statistical analyses were performed using SYSTAT13 (Systat Software, Inc., Chicago).

## Results

### Characterization of Natural English

**Figure 1:**
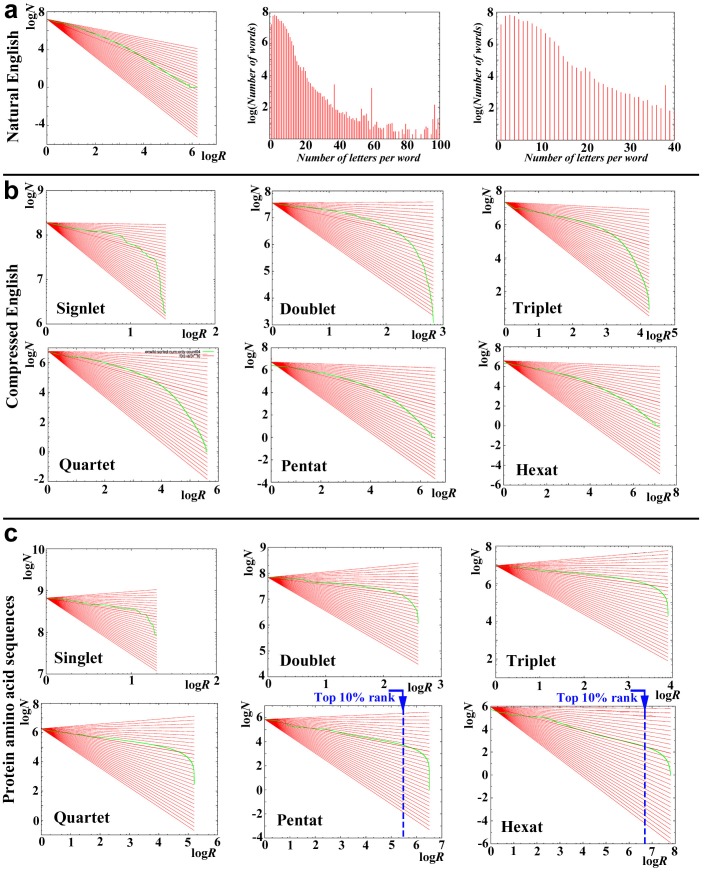
Distributions of English letters and protein amino acid sequences. The relationships between rank (*R*) and number (frequency) of words (*N*) are shown in the log-log plots. The background red lines indicate *Y* = *A – bX*, where *b* varies with an interval of 0.05. The thick red line indicates the best-fit least squares line. The green dots and lines indicate the observed results. (**a**) Natural English, following Zipf's law (left), and distribution of English word lengths (middle and right). The word lengths peak at 3. (**b**) Compressed English. (**c**) Protein amino acid sequences.

We first examined current natural English in reference to Zipf's law (or power law) using a web version of an encyclopedia, Wikipedia. The log-log plot of rank (*R*) in the *X*-axis and occurrence (*N*) in the *Y*-axis largely exhibited a nearly straight line within a linear range of 2.7–5.0 in *X*-axis (more accurately 10^2.7^–10^5.0^, but expressed as such just for convenience; linear width was thus 2.3) with an exponent *b* of 0.79 and a correlation coefficient *r* of 0.59 (Figure 1a, left; Table 1). A linear range in *Y*-axis was 1.3–5.0, and thus its linear width was 3.7. These linear ranges in *X*- and *Y*-axes spanned more than two orders of magnitude, indicating a scale-free nature of the English word distribution. We noted that the word length peaks at 3 and gradually declines as the length increases in natural English (Figure 1a, middle and right).

**Table 1:** *a*, *b*, and *r* values for English and proteins.

Letter Sequences	*a*	*b*	*r*
***Natural English***	1.8×10^7^	0.79	0.59
***Compressed English***			
Singlet	1.9×10^8^	0.53	0.77
Doublet	3.6×10^7^	0.49	0.83
Triplet	2.2×10^7^	0.60	0.74
Quartet	6.0×10^6^	0.54	0.80
Pentat	5.3×10^6^	0.58	0.74
Hexat	3.9×10^6^	0.58	0.73
***Proteins (nr-aa)***			
Singlet	6.6×10^8^	0.34	0.85
Doublet	6.9×10^7^	0.29	0.93
Triplet	9.1×10^6^	0.30	0.96
Quartet	7.3×10^5^	0.41	0.94
Pentat	8.3×10^5^	0.52	0.83

### Characterization of Compressed English

In the case of protein sequences, there is no space between words, as in the Japanese language, and we have no way to determine word gaps in proteins. Thus, we next examined no-space “compressed” English sentences to see if compressed English can retain the characteristics of original English. For simplicity, we assumed in this analysis that the word length (i.e., the number of letters in a word) is uniform. In other words, we assumed that all words have an identical number of letters (*n* = 1, 2, …, 6), similar to the triplet genetic code. But we counted all possible combinations of words by conceptually sliding a word window one by one over letter sequences. That is, we examined all “reading frames” simultaneously.

**Figure 2:**
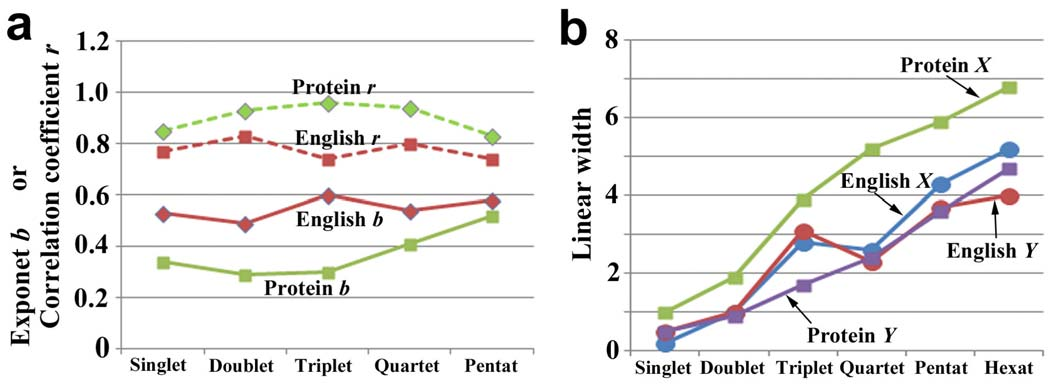
The exponent and linear widths of compressed English and proteins in the rank-frequency plot. (**a**) The exponent *b* and correlation coefficient *r* in compressed English and proteins. (**b**) The linear width in compressed English and proteins both in *X*- and *Y*-axes.

In compressed English, the log-log plots of rank and occurrence exhibited the exponents that were smaller than that of natural English but their correlation coefficients were larger (Figures 1b, 2a; Table 1). The largest exponent *b* was 0.60 in triplet, but those of pentat and hexat, 0.58, were comparably large (Figure 2a; Table 1). Notably, their linear ranges and widths in pentat and hexat were larger than those of natural English (Figure 2b; Table 3). Discriminant *R* values were positive for triplet, quartet, pentat, and hexat (Table 2). These results demonstrate that compressed English favors a power law over an exponential when it is considered to be composed of 3-, 4-, 5-, or 6-letter words.

**Table 2:** Discriminant *R* values for English and proteins using powerlaw.4.1.

Letter Sequences	All rank used		Top 50% ranks		Top 10% ranks	
	*R*	*p*-value	*R*	*p*-value	*R*	*p*-value
***Natural English***	5.8×10^6^	0.0	5.8×10^6^	0.0	4.4×10^5^	0.0
***Compressed English***						
Singlet	–6.0×10^1^	3.1×10^–47^	–1.5	0.20	6.8×10^–3^	0.97
Doublet	–5.1×10^2^	1.4×10^–23^	–3.7×10^1^	0.0010	–1.9	0.44
Triplet	2.5×10^4^	0.0	5.9×10^3^	4.9×10^–134^	7.5×10^1^	0.090
Quartet	1.3×10^6^	0.0	4.5×10^5^	0.0	2.2×10^4^	0.0
Pentat	1.3×10^7^	0.0	5.0×10^6^	0.0	4.3×10^5^	0.0
Hexat	4.8×10^7^	0.0	2.4×10^7^	0.0	2.2×10^6^	0.0
***Proteins (nr-aa)***						
Singlet	–5.6×10^1^	0.0	–1.1×10^–1^	0.80	1.5×10^–3^	0.99
Doublet	–1.0×10^4^	0.0	–1.8×10^1^	5.7×10^–8^	7.6×10^–2^	0.92
Triplet	–1.8×10^4^	0.0	–4.1×10^2^	3.4×10^–70^	–7.2	0.31
Quartet	–2.8×10^5^	0.0	–6.3×10^3^	0.0	1.5×10^1^	0.78
Pentat	–3.9×10^6^	0.0	–3.3×10^4^	1.8×10^–138^	1.8×10^4^	7.9×10^–226^

**Table 3:** The linear ranges and linear widths of compressed English and protein amino acid sequences.

SCS (word)	Singlet	Doublet	Triplet	Quartet	Pentat	Hexat
*N* of constituent aa*	1	2	3	4	5	6
**English**						
Linear range *X*	0.9–1.1	1.5–2.5	0.7–3.5	1.9–4.5	0.7–5.0	0.5–5.7
Linear width *X*	0.2	1.0	2.8	2.6	4.3	5.2
Linear range *Y*	7.4–7.9	6.0–7.0	3.7–6.8	3.7–6.0	2.3–6.0	2.0–6.0
Linear width *Y*	0.5	1.0	3.1	2.3	3.7	4.0
**Protein**						
Linear range *X*	0.3–1.3	0.6–2.5	0.0–3.9	0.0–5.2	0.6–6.5	1.1–7.9
Linear width *X*	1.0	1.9	3.9	5.2	5.9	6.8
Linear range *Y*	8.3–8.8	6.9–7.8	5.3–7.0	3.9–6.3	2.3–5.9	0.3–5.0
Linear width *Y*	0.5	0.9	1.7	2.4	3.6	4.7

Note: linear range of natural English (*X, Y*) = (2.7–5.0, 1.3–5.0); linear width of natural English (*X, Y*) = (2.3, 3.7).

* aa: amino acid.

The 3-letter word (triplet) system had the largest exponent among the *n*-letter word systems examined here. The linear widths in both *X*- and *Y*-axes peaked at the 3-letter word (triplet) system, although the 5- and 6-letter word (pentat and hexat) systems showed larger linear widths. These results did not contradict the fact that natural English (without compression) has mode and mean values of letter lengths of 3 and 4.8, respectively.

### Characterization of Protein Amino Acid Sequences

The rank-frequency plots of protein sequences using the nr-aa database with 1- to 5-amino-acid lengths (singlet, doublet, triplet, quartet, and pentat) showed exponents that were smaller than 1.0 (Figure 1c, 2a; Table 1) but exhibited straight lines in wider ranges than those of natural and compressed English (Figures 1c, 2b; Table 3). Both the exponent and linear range gradually increased, as the amino acid length of the system increased (Figure 2a, b). We were unable to detect any peaks of exponent and linear range, in contrast to compressed English. Notably in proteins, low-rank SCSs appeared to be highly deviated from the straight line in the systems of any word lengths (Figure 1c). Large linear ranges, relatively small exponents, and deviation of low-rank samples were three features that may characterize the protein distributions. Among them, large linear range is one of the requirements for a power-law distribution [29], but small exponent is not. Overall, the protein distributions are likely different from a simple power-law distribution.

**Figure 3:**
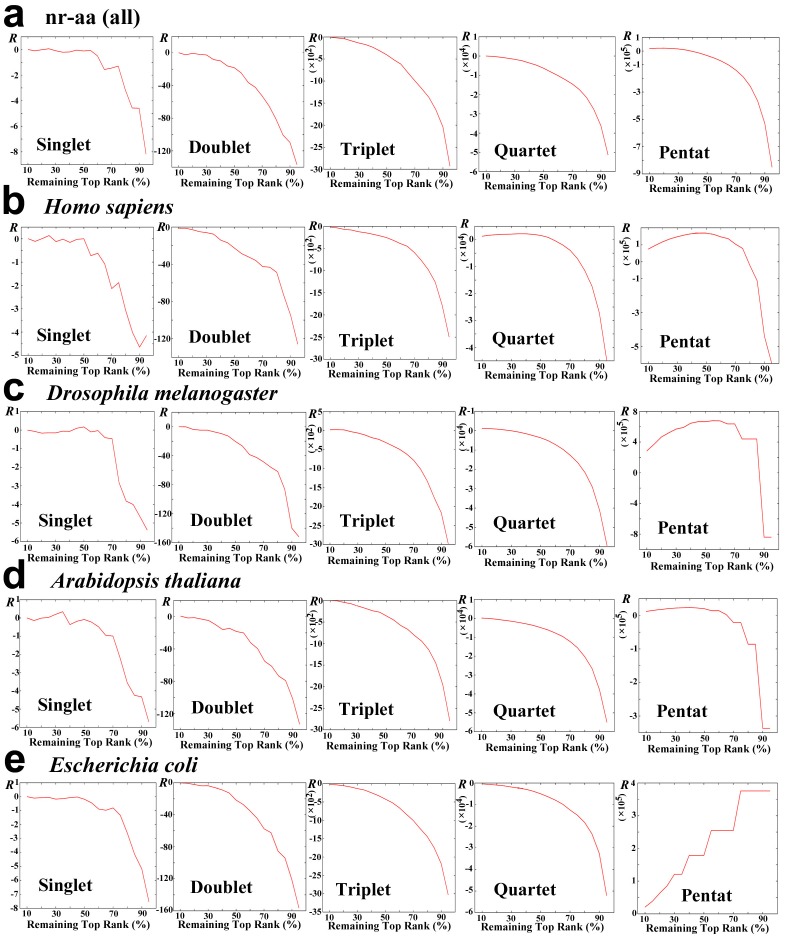
Relationship between remaining top rank sample (%) and discriminant *R* value. (a) nr-aa (all). (b) *H. sapiens*. (c) *D. melanogaster*. (d) *A. thaliana*. (*e*) *E. coli*.

It is highly likely that deviation of low-rank samples serves as a noise to prevent the protein SCSs to exhibit a power-law distribution. As expected, despite the large linear ranges, all *n*-aa SCS systems showed highly negative discriminant *R* values (Table 2). We also obtained discriminant *R* values for protein samples with top 50% ranks and top 10% ranks (Table 2). Use of top 10% ranks made discriminant *R* values positive, although only the pentat system had significantly low *p*-values (Table 2). The relationship between the remaining top rank samples (%) and discriminant *R* value indicated a strong contribution of the low-rank samples to the negativity of discriminant *R* value (Figure 3a).

We then examined behavior of natural English by obtaining discriminant *R* value using a program called “powerlaw”. Although the exponent of natural English did not reach 1.0 as discussed above, discriminant *R* value was highly positive (Table 2), indicating that natural English favors a power law over an exponential.

We conclude that the low-rank samples of protein amino acid sequences do not follow a power-law distribution, but the high-rank samples tend to exhibit a scale-free distribution with reasonably large linear ranges of both *X*- and *Y*- axes.

### Species-dependent Variations of Protein Distribution

**Figure 4:**
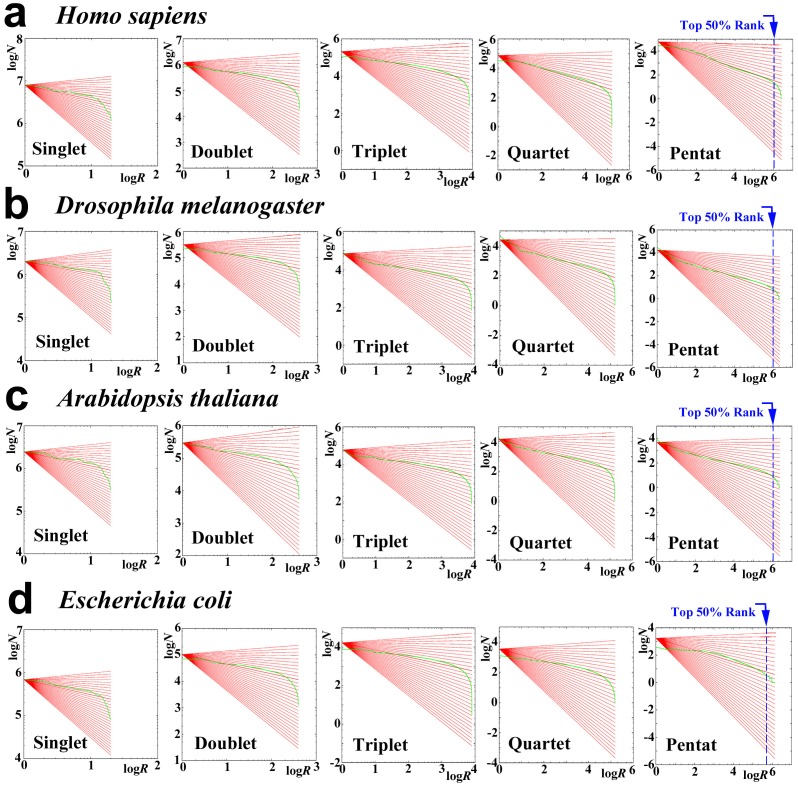
The rank-frequency relationship of proteins in four model organisms. Boundaries at the top 50% rank samples were indicated by blue dotted line. (**a**) *H. sapiens*. (**b**) *D. melanogaster.* (**c**) *A. thaliana*. (**d**) *E. coli*.

**Figure 5:**
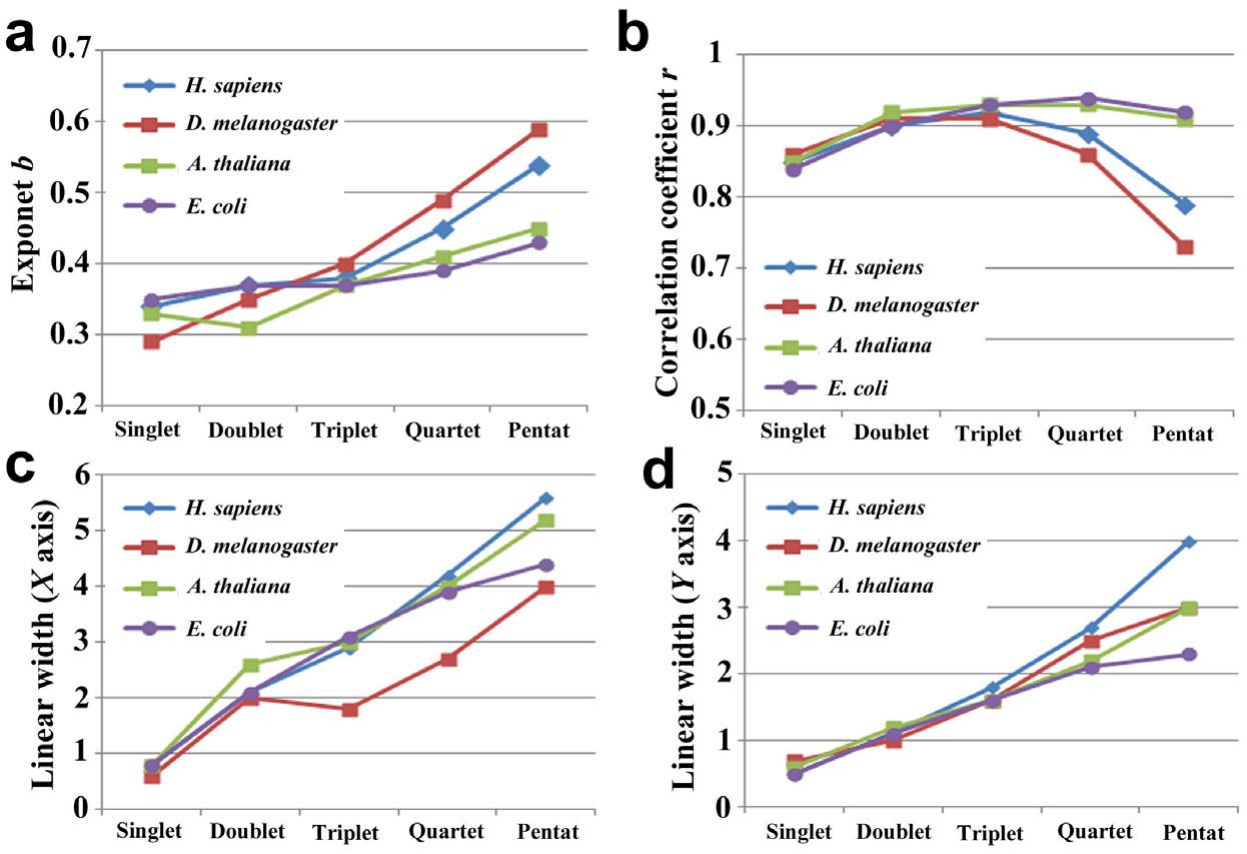
The exponents and linear widths of proteins from four model organisms in the rank-frequency plots. (**a**) The exponent *b*. (**b**) The correlation coefficient *r*. (**c**) The linear width of *X*-axis. (**d**) Linear width of *Y*-axis.

We next used the human (*Homo sapiens*), fly (*Drosophila melanogaster*), plant (*Arabidopsis thaliana*), and bacterium (*Escherichia coli*) proteins to examine whether the distribution characteristics that were observed in the nr-aa database are present in a species-dependent or species-independent fashion (Figure 4). The systems of these species showed very similar rank-frequency distributions to one another and to those of the nr-aa database; large linear ranges, relatively small exponents, and deviation of low rank samples were all observed (Figures 4, 5; Tables 4, 5). For example, the exponents, correlation coefficients, and liner widths of the pentat systems of the human and fly proteins (Figure 5; Tables 4, 5) were comparable to those of the compressed English (Figure 2; Tables 1, 3). On the other hand, we also observed differences between species (Figure 5; Tables 4, 5). For example, the human system showed relatively large linear widths both in *X*- and *Y*-axes and relatively high exponents among the four species that were examined.

**Table 4:** *a*, *b*, and *r* values for species-specific proteins.

Letter Sequences	*a*	*b*	*r*
***Homo sapiens***			
Singlet	8.0×10^6^	0.34	0.85
Doublet	1.2×10^6^	0.37	0.90
Triplet	2.1×10^5^	0.38	0.92
Quartet	7.4×10^4^	0.45	0.89
Pentat	6.1×10^4^	0.54	0.79
***Drosophila melanogaster***			
Singlet	2.0×10^6^	0.29	0.86
Doublet	3.2×10^5^	0.35	0.91
Triplet	6.7×10^4^	0.40	0.91
Quartet	2.6×10^4^	0.49	0.86
Pentat	1.8×10^4^	0.59	0.73
***Arabidopsis thaliana***			
Singlet	2.5×10^6^	0.33	0.85
Doublet	3.0×10^5^	0.31	0.92
Triplet	5.9×10^4^	0.37	0.93
Quartet	1.5×10^4^	0.41	0.93
Pentat	4.8×10^3^	0.45	0.91
***Escherichia coli***			
Singlet	6.9×10^5^	0.35	0.84
Doublet	1.0×10^5^	0.37	0.90
Triplet	1.7×10^4^	0.37	0.93
Quartet	3.44×10^3^	0.39	0.94
Pentat	1.7×10^3^	0.43	0.92

**Table 5:** The linear ranges and linear widths of amino acid sequences of species-specific proteins.

SCS (word)	Singlet	Doublet	Triplet	Quartet	Pentat
*N* of constituent aa*	1	2	3	4	5
***Homo sapiens***					
Linear range *X*	0.3–1.1	0.5–2.6	0.9–3.8	0.8–5.0	0.7–6.3
Linear width *X*	0.8	2.1	2.9	4.2	5.6
Linear range *Y*	6.3–6.8	4.7–5.8	3.0–4.8	1.8–4.5	0.3–4.3
Linear width *Y*	0.5	1.1	1.8	2.7	4.0
***Drosophila melanogaster***					
Linear range *X*	0.5–1.1	0.5–2.5	1.0–2.8	1.3–5.0	1.8–6.3
Linear width *X*	0.6	2.0	1.8	3.7	4.5
Linear range *Y*	5.8–6.5	4.3–5.3	2.7–4.3	1.0–3.5	0.0–3.0
Linear range *Y*	0.7	1.0	1.6	2.5	3.0
***Arabidopsis thaliana***					
Linear range *X*	0.3–1.1	0.0–2.6	0.8–3.8	1.0–5.0	1.1–6.3
Linear width *X*	0.8	2.6	3.0	4.0	5.2
Linear range *Y*	5.7–6.3	4.3–5.5	2.5–4.3	1.3–3.5	0.0–3.0
Linear width *Y*	0.6	1.2	1.6	2.2	3.0
***Escherichia coli***					
Linear range *X*	0.3–1.1	0.5–2.6	0.8–3.9	1.1–5.0	1.6–6.0
Linear width *X*	0.8	2.1	3.1	3.9	4.4
Linear range *Y*	5.3–5.8	3.7–4.8	2.2–3.8	0.8–2.9	0.0–2.3
Linear width *Y*	0.5	1.1	1.6	2.1	2.3

* aa: amino acid.

We then tested if these species-specific proteins favor a power-law distribution over an exponential. Discriminant *R* values were all negative except the pentat system of *E. coli*, indicating that they did not favor a power-law distribution with the exception of *E. coli* (Table 6). This could be due to the low-rank samples that contribute to deformation of the distributions. When only top 75% or 50% samples were used, positive discriminant *R*s were obtained for the pentat systems of most species (Table 6). We next checked relationship between the remaining top rank samples (%) and discriminant *R* value (Figure 3b-e). From the singlet to quartet systems, more elimination resulted in higher discriminant *R* values, but the maximum discriminant *R* values scarcely exceeded zero. However, in the pentat systems, elimination of up to about 50% resulted in the positive discriminant *R* values in *H. sapiens*, *D. melanogaster*, and *A. thaliana* (Figure 3b-d). Exception was again *E. coli*, in which the elimination process caused decrease of discriminant *R* values (Figure 3e).

**Table 6:** Discriminant *R* values for species-specific proteins using powerlaw. 4.1.

Letter Sequences	All rank used		Top 75% ranks		Top 50% ranks	
	*R*	*p*-value	*R*	*p*-value	*R*	*p*-value
***Homo sapiens***						
Singlet	–5.2×10^1^	0.0	–1.9	1.8×10^–2^	–1.1×10^–3^	1.00
Doublet	–9.0×10^2^	0.0	–4.3×10^1^	3.4×10^–12^	–2.3×10^1^	9.4×10^–11^
Triplet	–1.4×10^4^	0.0	–7.7×10^2^	1.5×10^–91^	–2.5×10^2^	3.0×10^–23^
Quartet	–1.8×10^5^	0.0	–7.0×10^3^	5.2×10^–77^	1.6×10^3^	6.0 ×10^–8^
Pentat	–6.0×10^5^	0.0	7.8×10^4^	6.1×10^–53^	1.7×10^5^	0.0
***Drosophila melanogaster***						
Singlet	–5.0×10^1^	0.0	–2.8	1.6×10^–6^	1.7×10^–1^	0.65
Doublet	–8.5×10^2^	0.0	–5.5×10^1^	1.6×10^–22^	–2.0×10^1^	2.0×10^–11^
Triplet	–1.3×10^4^	0.0	–1.0×10^3^	1.4×10^–122^	–3.1×10^2^	7.3×10^–19^
Quartet	–1.5×10^5^	0.0	–1.6×10^4^	4.8×10^–288^	–3.7×10^3^	7.6×10^–21^
Pentat	–8.4×10^4^	2.3×10^–196^	4.4×10^4^	1.6×10^–66^	6.7×10^4^	1.4×10^–234^
***Arabidopsis thaliana***						
Singlet	–5.0×10^1^	0.0	–2.2	1.0×10^–3^	–8.5×10^–2^	0.84
Doublet	–8.6×10^2^	0.0	–6.1×10^1^	8.5×10^–25^	–1.8×10^1^	5.5×10^–7^
Triplet	–1.3×10^4^	0.0	–9.4×10^2^	9.1×10^–132^	–3.6×10^2^	4.5×10^–44^
Quartet	–1.5×10^5^	0.0	–1.5×10^4^	0.0	–4.6×10^3^	7.3×10^–141^
Pentat	–3.4×10^5^	0.0	–2.1×10^4^	4.9×10^–40^	2.0×10^4^	5.0×10^–52^
***Escherichia coli***						
Singlet	–4.8×10^1^	0.0	–1.3	5.7×10^–2^	–1.8×10^–1^	0.70
Doublet	–7.9×10^2^	0.0	–6.2×10^1^	3.3×10^–24^	–2.2×10^1^	1.2×10^–11^
Triplet	–1.1×10^4^	0.0	–1.2×10^3^	1.7×10^–232^	–4.2×10^2^	2.8×10^–77^
Quartet	–7.4×10^4^	0.0	–1.5×10^4^	0.0	–5.0×10^3^	1.2×10^–233^
Pentat	3.8×10^5^	0.0	3.7×10^5^	0.0	1.8×10^5^	0.0

We conclude that the word distributions are largely similar among species, but some species-specific features are also present, which are more prominent in the pentat systems. In analogy to natural languages, species-specific protein languages may be considered “dialects”.

### Relative Availability Score Matches Sequence Motifs

We next explored whether we can identify functionally important short sequences, or “key words”, in proteins. We anticipated that key words would be at least partially equivalent to sequence motifs in proteins. Sequence motifs are defined in a sequence context (not in a structural context) as small stretches of amino acids that are highly conserved among related proteins. Using a collection of 1,641 representative proteins, we examined the possible relationship, if any, between the high-availability sites and known sequence motifs.

**Figure 6:**
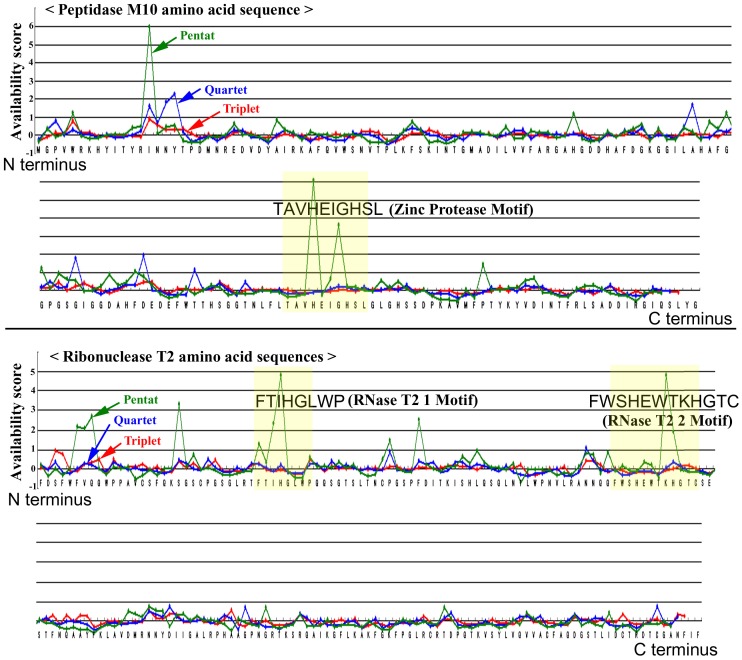
The availability plots of two examples of amino acid sequences of proteins, peptidase M10 (top) and ribonuclease T2 (bottom). *X*-and *Y*-axes indicate protein amino acid sequence and availability score, respectively.

Is it possible for the availability score to specify motifs efficiently in a given amino acid sequence? We found 521 motifs in 397 proteins out of 1,641 using the PROSITE search. Visual inspection of these motifs with availability plots showed that some motifs were “captured” nicely by high-availability sites using the pentat system and that non-motif regions showed much lower availability scores (Figure 6).

**Figure 7:**
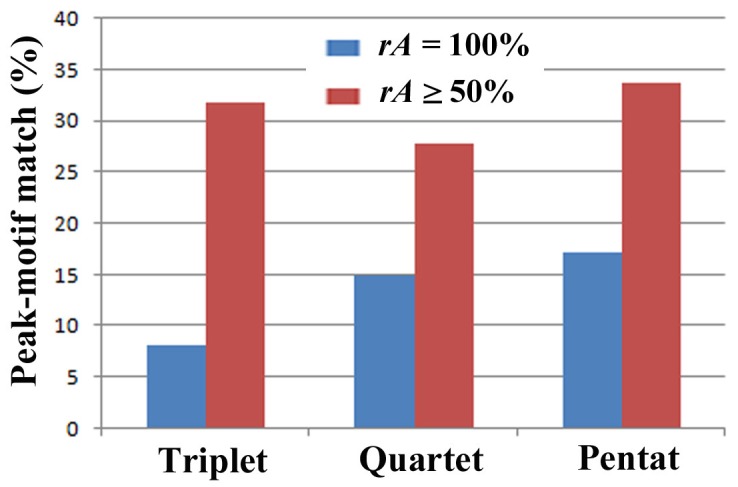
The percentages of correspondence between sequence motifs and SCSs (i.e., triplets, quartets, and pentats). The blue bars indicate *rA* = 100%, whereas the red bars indicate *rA*≥50%.

In identifying motifs based on availability scores, the relative values in a given protein are more important than an arbitrary threshold value. We thus calculated the relative availability score (*rA*) for a given protein: it is derived from the maximum availability score that was set to 100%, with the other scores being proportionally calculated. We found that 17.1% of these motifs can be identified by the positions of pentats, with *rA* = 100% (Figure 7). That is, the probability that the highest pentat peak in a given protein matches a motif is 17.1%. Similarly, when we use the arbitrary threshold for pentats, *rA*≥50%, these peaks match motifs in 33.5% of the cases (Figure 5).

### Sequence Motifs are Rich in High-availability Sites

Among these 397 motif-containing proteins, pentats with *A*≥3 occupied 9.5% of all possible pentats in these proteins. To examine whether motifs contain high-availability sites more than the entire proteins do, we calculated the percentage of pentats with *A*≥3, which was 12.9%. This result indicates a 1.4-fold enrichment of high-availability sites in motifs. Similarly, sequence motifs occupied 5.4% of the entire amino acids of 397 proteins. To examine whether high-availability sites (pentat *A*≥3) are enriched in motifs, we calculated the percentage of motifs in the high-availability sites, which was 10.5%. This result indicates a 1.9-fold enrichment of motifs in high-availability sites (pentat *A*≥3).

**Figure 8:**
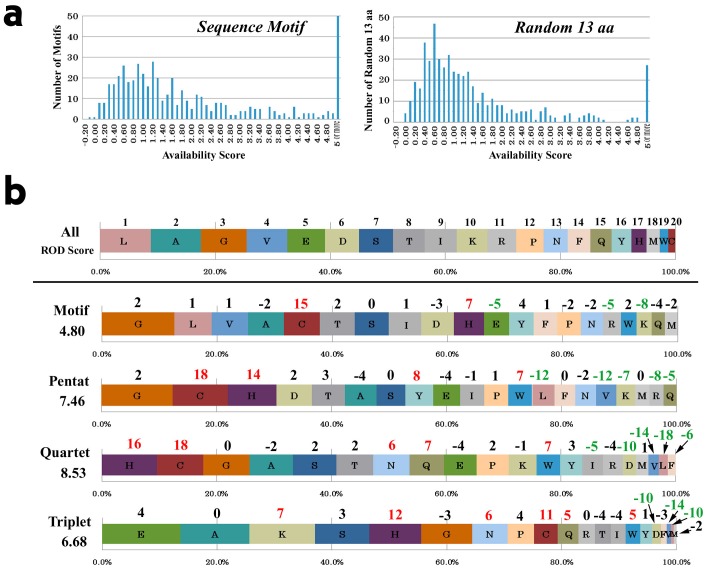
The characterization of high-availability sites within motifs. (***a***) The distribution of the pentat availability scores within motifs and random fragments. (***b***) The rank order analysis of the amino acid composition of full sequences, motifs, and high-availability areas of pentats, quartets, and triplets within motifs. The rank order distance (ROD) scores are shown beside the horizontal bars. The numbers at the top of each amino acid indicate the rank difference from the full sequences.

The availability scores of the pentats in motifs were compared with those of randomly specified amino acid regions taken from the same proteins (Figure 8a). The median values of motifs and random sequences were 1.20 and 0.60, respectively. The number of pentats with *A*≥5 is 50 in motifs and 25 in random sequences, respectively. The overall distribution patterns were significantly different as indicated by a Mann-Whitney *U*-test (*p*<0.0001).

### High-availability Sites within Motifs Contain Specific Amino Acids

We further characterized the high-availability triplets, quartets, and pentats (*A*≥3) within the 521 motifs identified above (Figure 8b). The ranking of the amino acid composition of the entire set of motifs was relatively similar to that of “all” sequences, with a relative order distance (ROD) score of 4.80. Exceptional amino acids with increased rank within motifs were C (cysteine; rank change score +15) and H (histidine; +7). The amino acid composition of high-availability sites (*A*≥3) within motifs showed more varied rankings. Hydrophobic amino acids, such as L (leucine), A (alanine), and V (valine), had high ranks both in all sequences and in motifs, but their rank orders decreased considerably in the high-availability pentats, quartets, and triplets. Instead, C (cysteine; +18 in pentats and quartets) and H (histidine; +14 in pentats and +16 in quartets) were prominent in the high-availability pentats and quartets. The rank order distance (ROD) scores were highest in the high-availability quartets, 8.53, and the high-availability pentats and triplets also showed relatively high ROD scores of 7.46 and 6.68, respectively. These analyses demonstrated that high-availability sites within motifs are distinct from other regions of motifs, suggesting that these sites may be functionally important.

## Discussion

In this study, we showed that at least a part of sequence information can be extracted by treating amino acid sequences of proteins as a natural language, i.e., current English. Our analysis of current English from Wikipedia is consistent with a recent mathematical study on the same data source [34]. That is, we confirmed that natural English most likely follows a power law. Furthermore, we showed that elimination of spaces that produced “compressed English” did not hamper a power-law distribution in the triplet, quartet, pentat, and hexat systems, mainly judging from discriminant *R* values (together with *p*-values) and linear widths both in *X*- and *Y*-axes in the log-log plot of rank and occurrence. Overall patterns of natural English and compressed English are similar to each other (see Figure 1a and 1b). Thus, we think that the compressed English retains important characteristics of natural English. This result may not be very surprising, because classical Latin literature was written without spaces before the seventh or eighth century [35]. Also, there are many languages that do not have spaces between words such as Japanese, Korean, and Chinese.

It appears that linear widths depend on the number of letters in words or SCSs to be examined. This is understandable, because a repertoire of SCSs increases dramatically when the number of letters in words or SCSs increases. Hence, it is not unexpected to find the fact that compressed English in the 6-letter (hexat) system scored best among the *n*-letter word systems examined here. This result does not immediately mean that English sentences are mostly composed of 6-letter words, because we did not perform analysis on 7-letter words or more. We detected peaks of the exponent and linear width at the 3-letter (triplet) system, which may indicate the average word length of natural English. However, we admit that this could be a simple coincidence.

We found that the frequency of SCSs of proteins showed a scale-free distribution only when low-rank tails are excluded. Although the power law behaviors are now found in many molecular biological systems [4], [36]–[41], Stumpf and Porter [29] proposed that, to conclusively show a scale-free nature of a given distribution, linearity must be obtained over at least two orders of magnitude both in *X*- and *Y*- axes in the log-log plot of rank and occurrence. We found that, in the quartet, pentat, and haxat protein systems, linear widths both in *X*- and *Y*-axes exceeded 2.0, and their linear widths are larger than those of the compressed English systems.

To be sure, not all studies affirmed the importance of the power law in analyzing the linguistic systems [42], and many claimed power-law systems, especially, biological network systems, may indeed be false-positive results, because they lack rigorous quantitative statistical analysis [29]–[32]. However, the recent mathematical analysis supported the traditional view of the usefulness of the power law [43]–[46]. Methodologically, the present study compared protein amino acid sequences to a natural language, English, which is known to follow a power law. To perform a comparative analysis, we used the python powerlaw program that statistically judges the feasibility of a given distribution to favor a power law over an exponential. Additionally, we directly examined linear width for a given distribution, which could serve as a relatively simple method to judge whether to have a scale-free nature. Considering that protein linear regions appeared to be larger than English, it is reasonable to think that the low-rank samples in proteins strongly contribute to the negativity of discriminant *R* values. Exclusion of the low-rank samples indeed made discriminant *R* values to be positive at least in the pentat system. Together, we can state that high-rank protein amino acid sequences tend to exhibit a scale-free distribution but low-rank tails do not, and this fact suggests that they may have language-like characteristics at least partly. The positive discriminant *R* value in the pentat system may be consistent with the fact that the length of secondary structures peaked at 5 or 6 amino acids [11], [12].

We observed a few characteristics that may be unique to protein distributions, which may be as important as similarity to English. First, the exponent is smaller than that of natural English despite wide ranges in linearity. However, this result is also observed in compressed English. Second, relatively large linear ranges are conspicuous in proteins in contrast to a loosely curved distribution both in natural and compressed English. Third, as discussed above, low-rank samples are highly deviated from a linear distribution. The biological significance of low-level samples in proteins is not known at this point. Fourth, in contrast to compressed English, we observed no peak in proteins; both exponent and linear width appear to increase gradually as the number of amino acids in SCSs increases. Examinations of heptats, octats, nonats, and so on are expected in the future.

The distribution patterns of four species that were examined here are largely similar to each other and to that of the nr-aa database, although there are some differences in the exponent and linear width among them. There seems no simple influence of database size among these four systems. However, the most important unique feature was found in the *E. coli* pentat system, where it showed positive discriminant *R* value without any elimination of low-rank samples. Whether this unique feature can be attributed to prokaryotic proteins remains elusive. It may be possible to find a species that exhibits a distribution that is more similar to the English distribution if other species are analyzed. Similarly, it may be possible to find a non-English language that exhibits a protein-type distribution if other natural languages are analyzed. Further studies are necessary to pursue these possibilities.

It is likely that protein amino acid sequences are linguistically more complex than human language texts [47], and thus we admit that there may be a technical limitation of our approach to decoding protein grammar. However, we identified putative “key words” (i.e., high-availability sites) in proteins, some of which corresponded nicely to sequence motifs. The correspondence of high-availability sites to motifs is much higher in the pentat system than in the quartet and triplet systems. However, if the working hypothesis is correct, proteins are likely to be written by words with different numbers of letters, and thus, the utility of quartets and triplets should not be disregarded entirely at this point.

Within motifs, the high-availability sites in the pentat system are richer in C (cysteine) and H (histidine), suggesting that high-availability sites may be the most important sites within motifs. We also examined the amino acid assignments [26] of motifs and those of high-availability sites within motifs, which confirmed that the high-availability sites within motifs are highly different from the entire motif sequences (data not shown). Furthermore, approximately 17% of the highest availability sites (*rA* = 100%) in the pentat system corresponded to known motifs. It is possible that the highest availability sites that had no correspondence to any known motifs may nonetheless be structurally and functionally important. It is also likely that this method disproportionately identifies certain groups of motifs.

Conventional motif identification and other amino acid sequence comparisons are largely dependent upon successful multiple alignments. Multiple alignments necessitate the sequence homology of relatively long stretches of sequences. It is apparent that sequence motifs are not enough to understand functionally important sites, considering that only 24.2% of all proteins examined (397 out of 1643) contained one or more motifs in our survey, despite the fact that all proteins would have functionally important sites. Our availability-based method is independent of extensive sequence homology among many samples. Rather, in the availability analysis, series of SCSs in a query protein sequence are compared with all known sequences in the nr-aa database. We anticipate that this word decoding approach can be complementary to the sequence alignment approach in predicting functional sites of unknown amino acid sequences. Use with other functional assignment programs, such as BLAST [3], PROSITE [33], and hydropathy plotting [48], may be fruitful.

For sequence comparisons, tuple analysis has been extensively performed [11]–[14], and a similar algorithm is used in BLAST to find relevant sequences with longer similarities [3]. However, the use of tuples, here called SCSs, has not been performed to find “key words” in conjunction with the linguistic analogy to current English. An interesting case was found in Sawada and Honda [49], in which structural diversity of short segments (identical to SCSs) that were obtained from the PDB was evaluated by a single-pass clustering algorithm. Notably, the structural diversity follows a power-law distribution [49], which may fortify the results of the present study in this paper.

It is well known that different amino acid sequences can form a similar three-dimensional structure and that an identical amino acid sequence can form alternative structures. This fact does not deny the presence of functional SCSs in proteins. In languages, different sentences can convey similar information, and an identical sentence can mean totally different things. Both in proteins and languages, the context is important in finalizing structures or meanings. This ambiguity or flexibility that was observed both in proteins and languages is further consistent with our working analogies between proteins and languages.

Overall, based on a working hypothesis that amino acid sequences are composed of SCSs that may be equivalent to words, we were able to detect important sites in proteins, although amino acid SCSs behave differently from English words in a few points. Exhaustive word identification efforts could eventually produce the dictionary of protein language. We speculate that words are positioned according to a set of rules, called grammar or idiomatic expressions, that can also be revealed by an availability-based method (a pilot program has already been built into the SCS package). These studies will contribute to protein engineering and rational drug design in the future.
